#  Multifunctional Polyvinyl Alcohol/Chitosan Composite Film Containing L-Cysteine and Pomegranate Peel Carbon Dots for Cherry Tomato Preservation 

**DOI:** 10.3390/foods15081316

**Published:** 2026-04-10

**Authors:** Limin Guo, Silong Jia, Linna Wang, Hesheng Wang, Qiyuan Feng, Xinyu Yang, Xi Lv, Yaqian Yang, Tian Li, Shaoying Zhang, Youwei Yu

**Affiliations:** College of Food Science, Shanxi Normal University, Taiyuan 030092, China; 223420013@sxnu.edu.cn (L.G.); 223420006@sxnu.edu.cn (S.J.); 223420011@sxnu.edu.cn (L.W.); wanghesheng2025@163.com (H.W.); 15713577244@163.com (Q.F.); 223420014@sxnu.edu.cn (X.Y.); 224420011@sxnu.edu.cn (X.L.); 224420009@sxnu.edu.cn (Y.Y.); 223420028@sxnu.edu.cn (T.L.)

**Keywords:** pomegranate peel carbon dots, L-cysteine, antimicrobial properties, UV shielding, food preservation

## Abstract

Currently, the resource wastage and safety hazards caused by fruit and vegetable spoilage are becoming increasingly prominent. Developing green, efficient, and non-toxic novel preservation materials has emerged as a hot spot in fruit and vegetable research. Based on this, this study utilized pomegranate peel as a raw material to prepare spherical multifunctional carbon dots (P-CDs) with an average particle size of 1.98 ± 0.58 nm through a one-step hydrothermal reaction. Subsequently, P-CDs were co-incorporated with L-cysteine (L-Cys) into a polyvinyl alcohol (PVA) and chitosan (CS) matrix to construct a novel composite coating material with combined antibacterial, antioxidant, and preservation functions. Experimental results demonstrate that P-CDs exhibit outstanding antioxidant activity and antibacterial performance. Compared to PVA/CS film, the P-CDs/L-Cys/PVA/CS film exhibited a 6.55 MPa increase in tensile strength and significantly enhanced thermal stability. Furthermore, the incorporation of P-CDs and L-Cys markedly boosted the PVA/CS film’s antioxidant activity (97% for ABTS; 85.69% for DPPH), antibacterial performance, and ultraviolet (UV) shielding capability. Coating cherry tomatoes with the P-CDs/L-cysteine/PVA/CS composite extended their shelf life by 6 days. This composite coating material exhibits preliminary biocompatibility and eco-friendly properties, aligning with green sustainable development needs and offering a novel potential solution for food preservation technology, while its practical applicability to food safety requires further comprehensive verification.

## 1. Introduction

With the growth of economic levels, global production and consumption of fruits and vegetables have also increased rapidly [1]. Key factors such as microbial contamination, ultraviolet radiation, and the physiological metabolism of agricultural products make them highly prone to decay and deterioration after harvest [2]. The application of fresh-keeping packaging materials plays a crucial role in maintaining the postharvest quality of fruits and vegetables and preventing spoilage. However, traditional preservation packaging has problems such as chemical residues, which endanger human health and cause secondary environmental pollution, making it difficult to meet the development needs of the modern green food industry [3]. As such, there has been considerable research focus in terms of creating safe, non-toxic and effective new packaging materials in the area of food preservation.

The concept of edible coatings has received much research effort due to its potential as a viable technique to prevent spoilage and preserve the quality of fruits and vegetables after harvest [4]. This coating has the ability to cover the surface of the fruits in a semi-permeable multifunctional protective layer against gaseous exchange and microbial contamination [5]. Biomacromolecules from natural sources, used as novel food packaging and preservation materials, are considered ideal alternatives to traditional petroleum-based plastic packaging due to their excellent biocompatibility, degradability, and functional tunability. Chitosan (CS) is a natural polysaccharide that has a linear amorphous structure, with many amino and hydroxyl groups on its molecular chain. These groups not only bestow an outstanding film-forming capacity and mechanical strength but can also permit modification of the composites with other materials by hydrogen bonds, ionic bonds, or covalent bonds. Protonated amino groups confer a positive charge, providing significant advantages in adsorbing anionic substances and in antimicrobial applications [6]. However, pure chitosan films suffer from drawbacks such as low mechanical strength and limited antimicrobial efficacy [7]. To enhance the overall performance of chitosan, researchers have combined it with antimicrobial agents or antioxidants. For instance, Ji et al. [8] determined that composite films prepared with peanut shell nanocellulose and chitosan had much better mechanical and barrier properties than native chitosan. Fan et al. [9] incorporated purslane extract into a chitosan–starch matrix to produce a composite film with outstanding antioxidant capacity. Polyvinyl alcohol (PVA) is a semi-crystalline polymer with several outstanding properties, including high chemical stability, excellent film-forming capabilities, high hydrophilicity, non-toxicity, and biodegradation [10]. The hydroxyl groups in its molecular chains can form hydrogen bonds with the amino groups on chitosan molecules [11]. Thus, the incorporation of chitosan and polyvinyl alcohol (PVA) can be used to improve the functionality of the membrane.

Antimicrobial properties of packaging reagents can be improved by incorporation of antimicrobial agents. However, traditional antimicrobial agents suffer from issues such as drug resistance, a narrow antimicrobial spectrum, and safety issues. Natural antimicrobial agents, such as plant essential oils and microbial metabolites, offer new solutions to these challenges. Nevertheless, essential oils are susceptible to high volatility, oxidation, and strong pungent odors [12]. Microbial metabolites also exhibit a limited antimicrobial scope; for instance, the anti-fungal activity of nisin is relatively weak, which makes it less effective in preserving fruits or bread [13]. L-cysteine (L-Cys) is a sulfur-containing hydrophilic amino acid possessing both a sulfhydryl group, a carboxyl group, and an amino group [14]. It is Generally Recognized as Safe (GRAS) for food use by the U.S. Food and Drug Administration [15]. While participating in chemical reactions, it also confers antioxidant and antimicrobial properties to composite films. Furthermore, L-cysteine serves as a precursor to hydrogen sulfide [16], which is a key signaling molecule in metabolic systems that inhibits ripening and senescence in various postharvest products. L-cysteine had also been used to preserve salmon [17], leafy greens [18], golden pears [19], plums [20], and lychees [21].

Carbon dots are zero-dimensional, quasi-spherical carbon-based nanomaterials smaller than 10 nm, featuring diverse chemical functional groups, excellent UV shielding properties, and significant antimicrobial and antioxidant capabilities [22]. It is interesting to note that the carbon dots produced using agricultural firm waste, like fruit peel, have high economic potential within the framework of green sustainable development and also show wide application perspectives in the field of food preservation. As an example, the composite film that was constructed by mixing cellulose nanocrystals of dialdehyde cellulose prepared using walnut green shells and carbon dots lengthened the shelf life of winter jujubes [23]. Likhar et al. [24] developed films from L-cysteine-modified carbon dots synthesized from Duea ching fruit, combined with chitosan and gelatin, for preserving sea bass filets. Pomegranate peel, rich in natural bioactive compounds like polyphenols and flavonoids [25], serves as an ideal raw material for green carbon dots. However, incorporating pomegranate peel carbon dots (P-CDs) alone into coating materials has limited effects on enhancing the film’s antimicrobial properties and mechanical performance. Inclusion of either P-CDs or L-cysteine in the film matrix has potential as an effective approach to enhancing the overall performance of the composite film, including its mechanical properties and antimicrobial action [17].

In summary, the following research gaps currently exist regarding the use of L-Cys and pomegranate peel carbon dots in active fresh-keeping packaging materials: (1) this study addresses the lack of clarity regarding the synergistic mechanism between natural biomass carbon dots and L-cysteine in PVA/CS films; (2) it resolves the challenge of achieving high-efficiency antibacterial properties, high antioxidant activity, and strong UV shielding simultaneously in single-function films; and (3) it expands the application pathways for the high-value utilization of agricultural waste in smart active packaging, providing a novel green solution for the long-term preservation of fruits and vegetables.

This study is the first to simultaneously incorporate pomegranate peel carbon nanodots and L-cysteine into a PVA/CS film matrix, resulting in the preparation of a PVA/CS/L-Cys/P-CD composite coating. By leveraging the interaction between the sulfhydryl groups of L-cysteine and the surface functional groups of carbon nanodots, the dispersion of carbon nanodots was improved, simultaneously achieving synergistic improvements in antioxidant, antibacterial, UV-shielding, and mechanical properties. The coating was applied to cherry tomatoes for preservation, and its preservation efficacy on fruits and vegetables, as well as its application potential in food preservation packaging, was evaluated, resulting in the development of a novel, green, multifunctional active packaging coating.

## 2. Materials and Methods

### 2.1. Materials

The Xuxi Fruit and Vegetable Store, located in Taiyuan City, Shanxi Province, was the place from which cherry tomatoes and pomegranates were obtained. We selected cherry tomatoes and pomegranates that were uniformly sized, moderately ripe, free from insect damage, and uncontaminated by microorganisms. Polyvinyl alcohol (PVA, Type 1799, alcoholysis degree 98–99%) and chitosan (CS, high viscosity, >400 MPa·s) were purchased from (Shanghai Aladdin Biochemical Technology Co., Ltd., Shanghai, China). L-cysteine (L-Cys, 99%, molecular weight: 121.16), 1,1-diphenyl-2-picrylhydrazyl (DPPH), 2,2′-azino-bis(3-ethylbenzothiazoline-6-sulfonic acid) (ABTS), and 2,6-dichloroindophenol (98%) were purchased from (Shanghai Yuanye Bio-Technology Co., Ltd., Shanghai, China). *Escherichia coli* (ATCC 11229), *Staphylococcus aureus* (CMCC 26003), and *Alternaria alternata* (BNCC 115062) were provided by the Fruit and Vegetable Storage and Processing Laboratory, College of Food Science, Shanxi Normal University. All types of reagents used are of analytical quality.

### 2.2. Preparation of P-CDs

**Figure 1:**
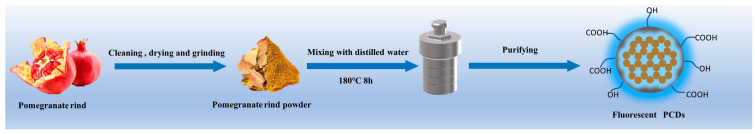
Schematic flowchart for hydrothermal synthesis of carbon dots from pomegranate peel.

The method of Pan et al. was used with slight alterations [26]. The pomegranate peel carbon dots (P-CDs) were produced through a one-step hydrothermal reaction, as the Figure 1 demonstrates. Fresh pomegranate peels were washed using distilled water, dried, and ground into powder using a mill. We prepared a 5 g mixture of pomegranate peel powder with 300 mL of distilled water, which was sonicated over a 30 min period. Then, the mixture was poured into a polytetrafluoroethylene polymethyl terminal fluoride (PTFE or Teflon) hydrothermal reactor. It was heated at 180 °C in an oven for 6 h. The mixture was centrifuged at 10,000 rpm and then allowed to settle until the mixture reached room temperature. A sample was filtered using a 0.22 μm membrane, after which the supernatant was collected. The filtrate was dialyzed next in distilled water over a 48 h period with a dialysis bag which had a molecular weight cut-off of 1000 Da; the dialysis solution was changed every 6 h. The powder of P-CDs was freeze-dried.

### 2.3. Characterization of P-CDs

#### 2.3.1. Transmission Electron Microscopy (TEM)

The solution comprised diluted P-CDs, and a sample was placed on a copper grid with an ultrathin carbon support film (200-mesh) under the infrared lamp. Transmission electron microscopy (200 kV field emission, JEOL-JEM F200, Tokyo, Japan) was used to view the microstructure of P-CDs. Finally, P-CD dimensions and particle size distribution were measured in ImageJ software (Fiji 2.3.0; ImageJ 1.53 k Wayne Rasband and Contributors, National Institute of Health, Bethesda, MD, USA).

#### 2.3.2. Ultraviolet–Visible Spectroscopy (UV)

The UV temperature of P-CDs was recorded in a dual-beam UV-Vis spectrophotometer (Evolution 220, Waltham, MA, USA). The range of the scanning wavelength was 200–700 nm.

#### 2.3.3. Phosphorescence Spectra (PL)

The phosphorescence properties of P-CDs were analyzed using a phosphorescence spectrophotometer (F98; Shanghai Lengguang, Shanghai, China). The photomultiplier tube voltage for both excitation and emission was set to 400 V, with a slit width of 5 nm.

#### 2.3.4. Fourier Transform Infrared Spectroscopy (FTIR)

The chemical functional groups of P-CDs were characterized by FTIR spectroscopy (Nicolet iS50, Thermo Fisher, Waltham, MA, USA) at 25 °C. The FTIR spectra were measured in the wavenumber range of 400–4000 cm^−1^ with a scan rate of 64 s.

#### 2.3.5. X-Ray Diffraction (XRD)

The crystal morphology of P-CDs was examined using an X-ray diffractometer (Ultima IV-185, Rigaku Corporation, Japan). P-CD powder was uniformly distributed onto an XRD sample holder and scanned over a range of 2–80° at a scanning speed of 5° per minute.

#### 2.3.6. X-Ray Photoelectron Spectroscopy (XPS)

XPS (K-alpha+, ThermoFisher, Waltham, MA, USA) was used to analyze the surface elemental composition of P-CDs. P-CD samples were placed in a special XPS sample holder, and a flat, uniform surface was ensured. The stage was subsequently dropped into the XPS instrument sample chamber. The excitation source was that of copper K-alpha radiation, and the range of the scanning angle was 5–40°. Once the high-resolution XPS scan was completed, the data from photoelectron spectroscopy was obtained and stored. The XPS data obtained were reported as binding energy in electron volts (eV).

#### 2.3.7. P-CD Cytotoxicity Assay

Cytotoxicity of P-CDs was tested using an approach by Wu et al. [27] in L929 mouse fibroblast cells. L929 cells were diluted to 1 × 10^5^ cells/mL and seeded into a 96-well plate. After 24 h of incubation to allow cells to fully adhere, the supernatant was removed. Next, 1000 μL of P-CD solutions of various levels (0.25, 0.5, 1, 2 mg/mL) were added and allowed to incubate for 24 h. The supernatant was then removed and placed in 100 μL of the CCK-8 solution. The OD was measured at 450 nm after 2 h. Formula (1) was used to obtain cell viability.

(1) $$\begin{array}{c} Cell\;viability\;\left(\%\right)=\frac{A_{1}}{A_{0}}\times 100 \end{array}$$

where A_1_ and A_0_ represent the absorbance values of the experimental group and control group, respectively.

#### 2.3.8. Hemolysis Assay

Following the method described by Cui et al. [28], the biocompatibility of P-CDs was evaluated using a hemolysis assay. In a simple trial, 4% mouse red blood cells were combined with P-CDs in the following concentrations of 0.25 mg/mL, 0.5 mg/mL, 1 mg/mL, and 2 mg/mL. The reaction mixture was allowed to incubate at 37 °C (3 h), and the reaction mixture was centrifuged to separate the supernatant and the absorbance at the wavelength 492 nm. The negative control was the incubation solution that was supplemented with PBS. An incubation solution with ultrapure water acted as the positive control. Hemolytic activity was calculated using formula (2):

(2) $$\begin{array}{c} Hemolysis\;\left(\%\right)=\frac{{OD}_{492nm\;P-CDs}-{OD}_{492nm\;blank}}{{OD}_{492nm\;ultrapure}-{OD}_{492nm\;blank}}\times 100 \end{array}$$

#### 2.3.9. Antioxidant Activity of P-CDs

The antioxidant effect of P-CDs was examined using one of the methods based on Xia et al.’s research [29]. DPPH and ABTS radical scavenging techniques were used to assess the antioxidant activity of P-CDs at different concentrations.

The ratio of the components of the final reaction mixture was 1 mL of P-CD solution and 4 mL of freshly prepared DPPH or ABTS solution. The absorbances at 517 nm (DPPH) and 734 nm (ABTS), respectively, were then recorded after 30 min of reaction in the dark. To determine the radical scavenging activity, the following formula was used, where A_0_ and A_1_ are the absorbance of blank and P-CD samples, respectively.

(3) $$\begin{array}{c} \mathrm{Free}\;radical\;scavenging\;activity\left(\%\right)=\frac{A_{0}-A_{1}}{A_{0}}\times 100 \end{array}$$

#### 2.3.10. Antibacterial Activity of P-CDs

The antimicrobial activity of P-CDs against *S. aureus* and *E. coli* was investigated using the MIC method and the agar diffusion method [30]. The MIC method involves diluting 100 μL of the P-CD solution into a series of concentrations, adding it to the liquid medium, and mixing it with a bacterial suspension of 1 × 10^6^ CFU/mL. After 24 h of incubation, changes in turbidity were observed. The concentration at which no bacterial growth was observed was defined as the minimum inhibitory concentration (MIC). We mixed 250 μL of the activated *S. aureus* and *E. coli* suspensions with 25 mL of the culture medium. Subsequently, we poured the mixture into the culture dish containing Oxford cups. After allowing the medium to cool and solidify, the Oxford cups were removed, and 100 μL of the P-CD solution was injected into each well. The diameter of the inhibition zones was monitored after incubation at 37 °C for 24 h.

### 2.4. Preparation of L-Cys/P-CDs/PVA/CS Composite Coatings and Films

**Scheme 1:**
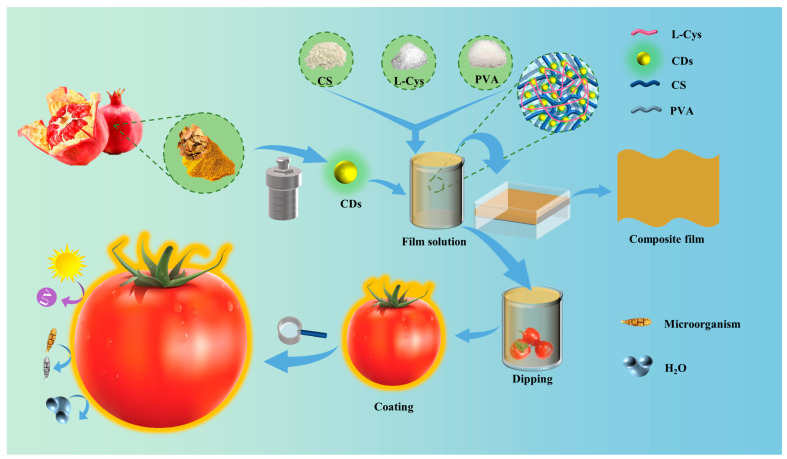
Flowchart of the coating material preparation and preservation procedure.

Composite PVA/CS films were prepared using solvent casting technology [31]. We added 2 g of PVA into 100 mL of distilled water and stirred at 90°C after 1 h to form a 2% PVA solution. Then, 1 g of CS was suspended in 100 mL of 1% glacial acetic acid and agitated at 60°C for 1 h to obtain a 1 per cent CS solution. A PVA/CS binary mixture was then formed by combining the two solutions, and the solution was added to 1 percent glycerol. Subsequently, 1% L-Cys, 1% P-CDs, and 1% L-Cys/1% P-CDs were added to the PVA/CS composite membrane solution, respectively. The solutions of the composite film were left in a 10 × 10 cm PTFE Petri dish and dried in a 45°C oven over a period of 48 h to obtain various composite films. The composite films were named PC (PVA/CS), PCL (L-Cys/PVA/CS), PCD (P-CDs/PVA/CS), and PCLC (L-Cys/P-CDs/PVA/CS). The process flow diagram for the composite films is shown in Scheme 1.

### 2.5. Characterization of Composite Films

#### 2.5.1. Scanning Electron Microscopy (SEM)

SEM (JSM-7500F, JEOL, Tokyo, Japan) was used to identify the microstructure of the thin film with an acceleration voltage of 5 kV. Prior to observation, fragments of the sample, brittle-fractured using liquid nitrogen, were mounted on conductive adhesive, gold-sputtered, and then placed in the sample chamber for examination.

#### 2.5.2. Ultraviolet–Visible Transmittance Measurement

Films were cut into 1 cm × 4 cm rectangles and placed against the inner wall of a cuvette. The transmittance of PC, PCL, PCD and PCLC films was recorded in the range of 200 nm to 800 nm on a dual-beam UV-Vis spectrophotometer (Evolution 220, Waltham, MA, USA).

#### 2.5.3. Fourier Transform Infrared Spectroscopy (FTIR)

A Fourier Transform Infrared Spectrometer (Is5, Thermo Fisher, Waltham, MA, USA) was employed to examine the chemical structure and molecular interactions of the films. Composite films were cut into 1 × 1 cm^2^ samples for analysis. Scan number: 32; scan range: 400–4000 cm^−1^; resolution: 4 cm^−1^.

#### 2.5.4. X-Ray Diffraction (XRD)

An X-ray diffractometer (Ultima IV-185, Rigaku, Tokyo, Japan) was used to determine the crystal structure of the composite film. Irradiation took place under Cu Kα radiation (0.15406 nm) with X-ray scanning conducted at 10 within the range of sample 2, which was taken to be 5–60° given the XRD pattern of P-CDs.

#### 2.5.5. Mechanical Properties

The mechanical characteristics of the films were tested with minor modifications to the procedure used by Zhang et al. [32]. The tensile strength (TS) and elongation at break (EAB) of the composite films were assessed using a texture analyzer (TA XT. plus SMS, Godalming, UK). The composite films were sliced into strips, 80 mm × 20 mm, and three parallel samples were used in each group. Test conditions were as follows: tensile speed of 50 mm/min at room temperature. Tensile strength (TS) and elongation at break (EAB) were calculated using Equations (3) and (4), respectively:

(4) $$\begin{array}{c} \mathrm{TS}\left(MPa\right)=\frac{F_{max}}{width\times thickness} \end{array}$$

(5) $$\begin{array}{c} \mathrm{EAB}\left(\%\right)=\frac{L-L_{0}}{L_{0}}\times 100 \end{array}$$

Fmax is the maximum tensile force (N); thickness and width are initial dimensions of the film (mm); L_0_ is the initial length of the specimen (mm); and L is the length of break of the specimen (mm).

The thickness of the film was measured with a digital micrometer (MDH-25MB, Mitutoyo, Shanghai, China). Three or more measurements (n= 3) were made at random points on each sample to obtain a representative average thickness value.

#### 2.5.6. Water Vapor Permeability (WVP) and Water Contact Angle (WCA)

The permeability of the film to water vapor was determined based on the method of Wang et al. [33]. First, anhydrous calcium chloride was placed in a beaker to fill approximately one-third of its height, ensuring the calcium chloride surface was level and did not contact the beaker rim. The film was then shut over the beaker. The test beaker was then placed into the desiccator at (25 ± 1) °C temperature, and a relative humidity of (75 ± 2) %. The desiccator also included a container of saturated sodium chloride solution to ensure a constantly humid environment. The test beaker was removed every 8 h for weighing until the weight remained constant, at which point weighing ceased. Each experimental group comprised 3 parallel samples, with calculations based on the average value. The process of water vapor permeability (WVP) is determined by the following formula:

(6) $$\begin{array}{c} \mathrm{WVP}=\frac{m\times L}{A\times t\times \Delta P} \end{array}$$

where Δm is the change in mass of the test cup over the period of the test (g); L is the thickness of the composite film (m); A is the effective test area of the film (m^2^); t is the time interval of the test (s); and 0 is the difference in the water vapor pressure on both sides of the film (Pa).

The water contact angle (WCA) of the composite film was tested on a contact angle tester (DSA25, Hangzhou, China) using the method of Cheng et al. [34]. The film was stored as 20 × 20 mm test samples, where 2 μL of distilled water was sprayed. During a time interval of 5 s, measurements of the changes in the water droplet were taken.

#### 2.5.7. Thermogravimetric Analysis (TGA)

The composite film was analyzed by thermogravimetric analysis with the help of a thermogravimetric analyzer (TGA 550, TA Instruments, New Castle, DE, USA). The conditions used when taking the test were as follows: temperatures between 25 °C and 600 °C, and heating at a rate of (N)/min in the presence of nitrogen.

#### 2.5.8. Antioxidant Activity of Composite Coatings

The basic method of Wang et al. [35] was used to determine the antioxidant activity of the composite film. The film was dissolved in 10 mL of distilled water using 30 mg of the film. The supernatant was then centrifuged at 6000 rpm for 10 min, and the DPPH working solution was added to the thoroughly mixed mixture and was left in the dark for 30 min. The solution absorbance of the mixture (B_1_), and the solution absorbance of DPPH (B_0_) were recorded at 517 nm. The radical scavenging activity was obtained by evaluating the DPPH radical at 517 nm, thereby calculating formula (7):

(7) $$\begin{array}{c} DPPH\;scavenging\;activity\left(\%\right)=\frac{B_{0}{-B}_{1}}{B_{0}}\times 100 \end{array}$$

We mixed equal volumes of ABTS solution (7.4 mM) with potassium persulfate solution (2.6 mM). The solution was then incubated at room temperature in the dark for 16 h. It was diluted with anhydrous ethanol until absorbance at 734 nm reached 0.7 (C_0_). Then, 30 mg of the membrane was cut and placed in 10 mL of deionized water; the supernatant was then centrifuged and mixed with the working solution and allowed to stand for 6 min. The absorbance of the solution was determined at 734 nm (C_1_) and the ABTS scavenging activity was calculated based on formula (8):

(8) $$\begin{array}{c} ABTS\;scavenging\;activity\left(\%\right)=\frac{C_{0}-C_{1}}{C_{0}}\times 100 \end{array}$$

#### 2.5.9. Antimicrobial Activity

The Oxford cup method was also used to test the inhibitory activity of varying composite membranes on Staphylococcus aureus and *Escherichia coli*. The specific procedure followed the method described in Section 2.3.10.

When carrying out the anti-fungal test, it was performed based on the approach outlined by Kyong et al. [36]. The agar diffusion method was used to determine the anti-fungal activity of the composite membrane on *Alternaria alternata*. *A. alternata* was cultured for 7 days before use, and a fungal disc was obtained from the edge of the colony using a sterile punch (9 mm diameter) for subsequent use. Two milliliters of culture medium were combined with 2 mL of composite membrane solution of different compositions and poured on Petri dishes. When the medium had solidified, the fungal discs were laid in the middle of each plate. The control group was added to plates on which the membrane solution was not added. The plates were incubated at 28 °C for 6 days, after which the colony diameters were measured to determine the anti-fungal effects of the coating solutions on *A. alternata*. The inhibition rate of *A. alternata* is calculated by the following formula:

(9) $$\begin{array}{c} Colony\;growth\;inhibition\;rate\;(\%)=\frac{D_{c}-D_{t}}{D_{c}}\times 100 \end{array}$$

Here, Dc represents the difference between the growth diameter of the control group and that of the treatment group, and Dt is the growth diameter of the treatment group, with the unit in millimeters.

### 2.6. Preservation Experiment of Composite Films on Cherry Tomatoes

We selected cherry tomatoes of uniform size, moderate hardness, free from mechanical damage and microbial contamination. They were immersed in a 0.01% sodium hypochlorite solution for 2 min, then rinsed thoroughly with distilled water, and the surface was air-dried to remove any remaining moisture. The cherry tomatoes were randomly divided into 5 groups, each containing 100 tomatoes. Each group was immersed in a PC, PCL, PCD, or PCLC composite film solution for 3 min, and then air-dried naturally. The control group comprised cherry tomatoes soaked in sterile water. The cherry tomatoes were kept at 25 °C with a humidity of 75% and treated.

#### 2.6.1. Weight Loss

The weighing method was used to determine the weight loss rate of cherry tomatoes. Cherry tomatoes were weighed every 3 days, and the rate at which weight was lost was determined using formula (10):

(10) $$\begin{array}{c} Weight\;Loss\;Rate\left(\%\right)=\frac{m_{0}-m_{t}}{m_{0}}\times 100 \end{array}$$

where m_0_ is the initial weight of cherry tomatoes, g; m_t_ is the weight of cherry tomatoes at the end of storage days, g.

#### 2.6.2. Rotting Rate

Visual inspection was carried out by establishing the rotting rate of cherry tomatoes. The decaying cherry tomatoes were counted after every three days. When fruits showed mold growth, soft rot or juice leakage, they were classified as rotten. The rotting rate was calculated using formula (11).

(11) $$\begin{array}{c} Rotting\;Rate\left(\%\right)=\frac{n_{t}}{n_{0}}\times 100 \end{array}$$

In this equation, n_0_ is the initial count of cherry tomatoes, and n_t_ is the count of rotten cherry tomatoes at the end of the days of storage.

#### 2.6.3. Hardness

The hardness of cherry tomatoes was measured using a texture analyzer (TA.XTC-20, Bosin Tech, Shanghai, China). Every three days, 10 tomatoes were randomly selected from each treatment. The P_2_ probe was punctured with a speed of 1 mm/s through the surface, and the strain was set at 80%. Hardness values were recorded in Newtons [1].

#### 2.6.4. Respiratory Intensity

A gas analyzer (F-940, Sunshine Medical, Beijing, China) was then used to measure the respiratory intensity of cherry tomatoes, with the results converted to the milligrams of carbon dioxide generated per hour divided by the kilograms of tomatoes present [37].

#### 2.6.5. Determination of Total Soluble Solids Content (TSS)

The total soluble solids were determined on a digital refractometer (PAL-1, ATAGO, Tokyo, Japan) and were given as a value in Brix.

#### 2.6.6. Vitamin C Content Determination

The vitamin C content in cherry tomatoes was used according to the strategy of Yu et al. [38]; the method used to measure it is the 2,6-dichlorophenolindophenol titration method. We took 10 g of the tomato tissue sample and homogenized the sample with 10 mL of 2% solution of oxalic acid, followed by dilution until 100 mL was left. We took 10 mL of the supernatant into a conical flask and titrated with 2,6-dichlorophenol–phenol solution until a pink endpoint was achieved and remained stable. The volume of titrant consumed was determined. The ascorbic acid content was obtained using the below formula: where K is the titration strength (mg/mL); V_1_ is the volume of 2,6-dichlorophenol solution used to complete the titration of the sample (mL); V_0_ is the volume of 2,6-dichlorophenol solution used to complete the titration of the blank (mL); V_s_ is the volume of the liquid sample to be used in the titration (mL); m is the mass of the sample to be titrated.

(12) $$\begin{array}{c} VC\;\left(mg/100\;g\right)=K\times \frac{V_{1}-V_{0}}{m}\times V_{s}\times 100 \end{array}$$

### 2.7. Statistical Analysis

All the experimental data were statistically analyzed using the SPSS 26 program. Findings are reported as the mean and standard deviation. Additional analysis was performed through one-way analysis of variance (ANOVA), and Duncan used the multiple range test to compare the results. A *p*-value of 0.05 was seen as statistically significant. Origin 2024 SRI software (Version 10.1.0.178) was used to collate the data and formulate a graph.

## 3. Results and Discussion

### 3.1. Characterization of P-CDs

#### 3.1.1. Morphology, Particle Size, and Optical Properties

**Figure 2:**
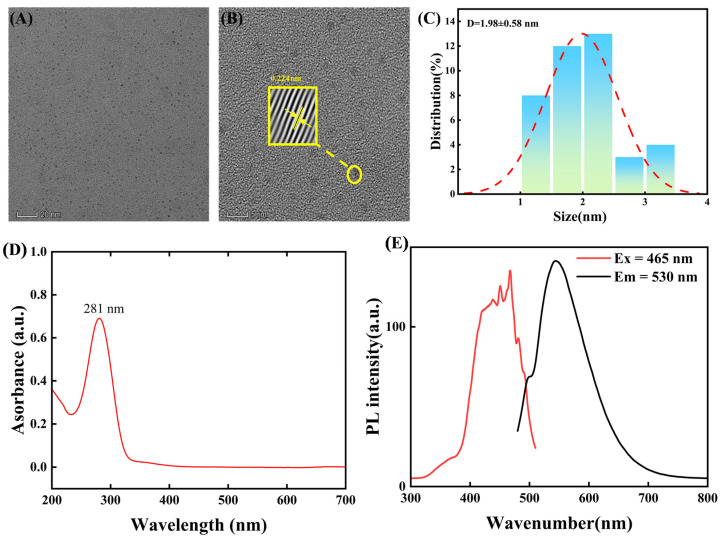
P-CD transmission electron microscopy image (**A**); lattice features (**B**); size distribution of P-CDs (**C**); UV–visible absorption spectrum (**D**); optimum excitation and maximum emission spectrum of P-CDs (**E**).

Figure 2A–C show TEM images and particle size distribution histograms of P-CDs. The TEM images reveal that the synthesized P-CDs exhibit excellent dispersion, appearing spherical or near-spherical with no significant agglomeration. Distinct lattice fringes are observed, with a lattice spacing of 0.224 nm. Statistical analysis of the average particle size of P-CDs was performed using ImageJ software, as shown in Figure 2C. The particle sizes of P-CDs were relatively uniform, with a distribution range primarily between 1.02 and 3.33 nm and an average particle size of 1.98 ± 0.58 nm. This aligns with the fundamental characteristics of carbon dots previously reported [39]. Additionally, Figure 2D,E present the UV-Vis absorption and photoluminescence spectra of the P-CDs solution. The UV absorption spectrum of P-CDs exhibits a prominent absorption peak at 281 nm (Figure 2D), which is attributed to n-π* transitions of -C=O or C–OH bonds and π-π* transitions of -C=N bonds [40]. Figure 2E shows that the frequency of the photoluminescence spectrum of P-CDs has a typical fluorescence beam with a significant output of emissions at 530 nm and 465 nm excitation. These properties systematically confirm the successful synthesis of carbon dots.

#### 3.1.2. Chemical Composition and Crystal Structure

**Figure 3:**
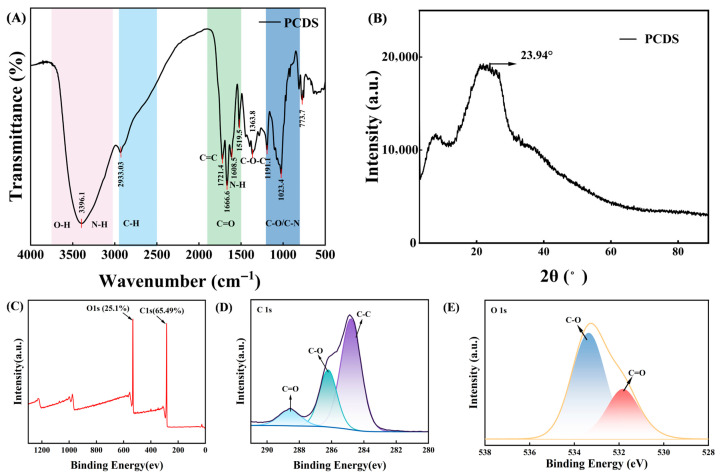
FTIR spectrum of P-CDs (**A**); XRD spectrum of P-CDs (**B**); XPS spectrum of P-CDs (**C**); high-resolution peak-fitted XPS spectra of C 1 s (**D**) and O 1 s (**E**). Values are expressed as mean ± standard deviation (*n* = 3).

FTIR was used to analyze the chemical functional groups of P-CDs, with the results presented in Figure 3A. The vibrations of -OH and -NH are observed at higher frequencies of 3396.1 cm^−1^ and 2933.03 cm^−1^, respectively, with the second peak caused by -C-H stretching [41]. The peak of 1721.4 cm^−1^ is related to the stretching vibration of -C=C; the peak of 1666.6 cm^−1^ is related to the stretching vibration of -C=O; the peak of 1608.5 cm^−1^ is related to the stretching vibration of -N-H; and the stretching vibration at 1363.8 cm^−1^ is related to -C-O-C [42]. Also, the stretching of -C-O and -C-N groups of acidic, alcoholic, epoxy, amine, and amide structures has been attributed to minor peaks in the range of 1300 to 820 cm^−1^. This outcome was in line with the research conducted by Eskalen et al. [43] in which these bonds were commonly observed on the surface of P-CDs.

Figure 3B displays the XRD pattern of P-CDs, with the center position of the graphite crystal plane at approximately 2θ = 23.94°. This pattern reveals the characteristic structure of carbon dot nanomaterials at 23.94°, similar to the results reported by Eskalen, Uruş, Kavgacı, Kalmış and Tahta [43]. The results indicate that P-CDs form a certain crystalline structure but primarily exist as disordered carbon nanoparticles, suggesting that P-CDs possess good surface activity [44].

Additional characterization with X-ray photoelectron spectrophotometers (XPSs) revealed that P-CDs formed the chemical elemental composition (as indicated in Figure 3C–E). C and O are the most common elements of P-CDs, with C being the element with the largest content of 65.49 and O being the most abundant element at 25.1. The C 1 s spectrum has three separate peaks of 284.8 eV, 286.2 eV and 288.6 eV, which are attributed to C-C, C-O and C=O of C-bindings, respectively. The highest value in the O 1 s spectrum, that is, 531.8 eV, belongs to the C=O bond, whereas 533.3 relates to the C-O bond. The FTIR analysis results are also similar to the XPS analysis results, and this shows that a variety of oxygen- and carbon-containing functional groups are present on the surface of the carbon dots. Through these functional groups, the carbon dots have a significant impact on their properties and applications [45].

#### 3.1.3. Cytotoxicity of P-CDs

**Figure 4:**
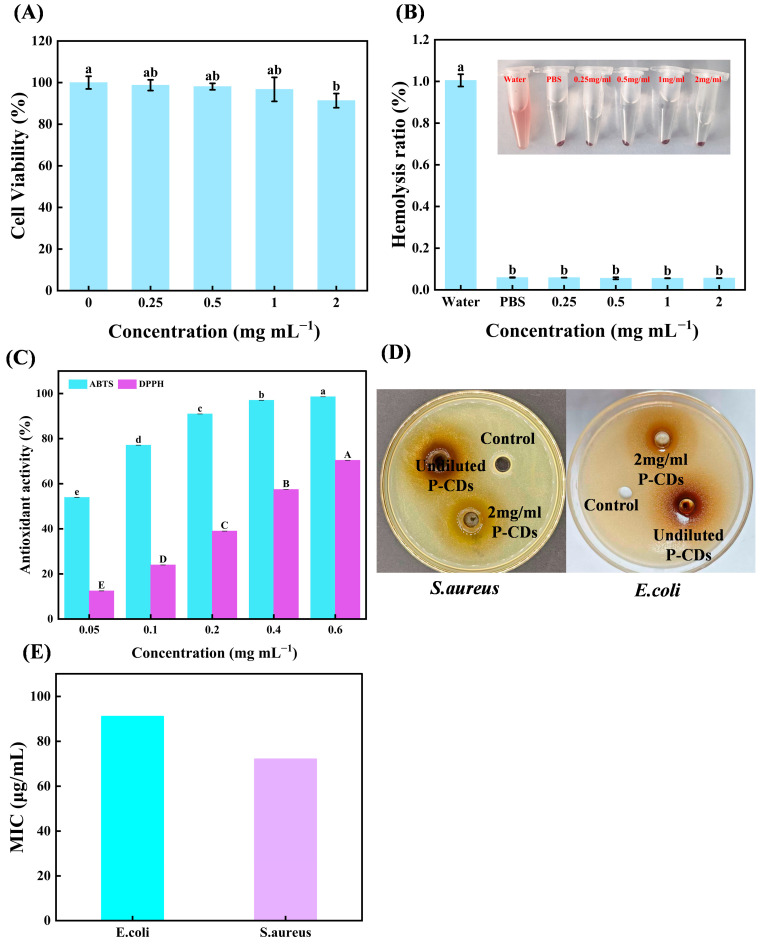
Effect of different concentrations of P-CDs on the survival rate of mouse L929 fibroblasts after 24 h of treatment (**A**); hemolysis of rat erythrocytes with different concentrations of P-CDs (**B**); antioxidant activity of P-CDs (**C**); antibacterial activity of P-CDs (**D**); MIC of P-CDs against bacteria (**E**). Values are expressed as mean ± standard deviation (*n* = 3). Different letters indicate significant differences at *p* < 0.05.

CCK-8 was used to measure the in vitro cytotoxicity of various concentrations of P-CDs with L929 mouse fibroblasts. The findings presented in Figure 4A reveal a minimal decline in the viability of cells as the level of P-CD concentration increased. Treatment of cells with 0.25, 0.5 and 1 mg/mL P-CDs did not show any significance in cell viability compared to the control group. Moreover, the highest cell viability, conditioned by the treatment with all P-CD concentrations, only demonstrates the preliminary biocompatibility of P-CDs and their applicability to food preservation bags [46].

#### 3.1.4. Hemolysis Assay of P-CDs

The biocompatibility of nanomaterials plays an extremely important role in the area of food preservation [47]. Hemolysis assay results showed that when comparing hemolytic activity with positive (red blood cells in ultrapure water) and negative controls (red blood cells in PBS), the hemolysis rate for all concentrations of P-CDs did not exceed 0.2%. Findings from Parvathy and Praseetha [48] indicated that a hemolysis rate below 5% is considered within the acceptable normal range, suggesting no disruption of the red blood cell membrane (Figure 4B). However, uniformly dispersed pink coloration was observed in red blood cells supplemented with ultrapure water, indicating complete hemolysis by ultrapure water. These phenomena stem from the carbon dots’ sub-10 nm size and multiple oxygen-containing functional groups. This chemical structure minimizes physicochemical interactions with red blood cells, preventing damage to their membranes. These results only indicate the preliminary biocompatibility of P-CDs, providing a basic experimental basis for their potential application in fresh-keeping materials, while their applicability to food safety needs further rigorous validation. The newly developed coating substance has shown potential for application in fruit and vegetable preservation.

#### 3.1.5. Antioxidant Activity of P-CDs

Oxidative reactions triggered by free radical generation are key factors causing food spoilage and nutrient degradation. Antioxidants, however, can scavenge free radicals, effectively slowing down the oxidative processes leading to food deterioration and spoilage [49]. The antioxidant performance of P-CDs was evaluated using ABTS and DPPH radical scavenging assays. Figure 4C indicates that the radical scavenging ability of P-CDs tended to increase with their concentrations. At 0.05 mg/mL, the DPPH radical scavenging rate reached only 12.48%, while the ABTS radical scavenging rate was 53.95%. At a concentration of 0.6 mg/mL, the scavenging efficiencies of P-CDs when considering the ABTS and DPPH radiological species were 98.58% and 70.38%, respectively. A rich number of organic functional groups on its surface endowed the antioxidant activity of P-CDs [50]. As an example, radicals in the phenolic hydroxyl group can undergo a reaction with free radicals to produce stable phenoxy radicals, and hence, radical chain reactions are ended. The formed phenoxy group is very stable, and with the conjugated effect, the likelihood of catalysts being related to new oxidative reactions is very limited [51].

Compared to DPPH, P-CDs exhibit higher antioxidant activity against ABTS. This may be attributed to P-CDs’ water solubility, which allows for greater dissolution in aqueous ABTS solutions than DPPH in methanol solutions [52]. This demonstrates the outstanding antioxidant properties of P-CDs, providing strong support for their practical application in food preservation.

#### 3.1.6. Antimicrobial Activity of P-CDs

The primary factor in food spoilage is the rapid proliferation of microorganisms. Substances with antimicrobial activity can effectively inhibit microbial growth, thereby preserving food quality and sensory characteristics [53]. Figure 4D,E show the inhibitory effects of P-CDs on *S. aureus* and *E. coli*, along with their respective MICs. The MICs for *E. coli* and *S. aureus* were 91 μg/mL and 72 μg/mL, respectively. Evidence suggests that P-CDs demonstrated significant antibacterial activity against *S. aureus* and *E. coli* (in comparison to the control condition). The inhibition zones for *S. aureus* measured 6.8 mm (2 mg/mL P-CDs) and 8.1 mm (undiluted P-CDs), while those for *E. coli* measured 5.9 mm (2 mg/mL P-CDs) and 6.2 mm (undiluted P-CDs). Regarding the antibacterial mechanism of carbon dots, Khan et al. [54] found that carbon dots generated reactive oxygen species (ROS) within cells, including ^∙^O_2_^-^, H_2_O_2_, OH·, and ^1^O_2_, which subsequently disrupt the cell wall, leading to cell lysis and death. On the other hand, Khan et al. [55] suggested an alternative antibacterial effect: cationic groups on the surface of carbon. This outcome also shows that the wall of P-CDs has a larger antibacterial zone against *S. aureus* than against *E. coli*. This is attributed to *S. aureus* (a Gram-positive bacterium), which possesses a thicker cell wall, making its membrane more susceptible to disruption by carbon dots. Secondly, the wall of E. coli (a Gram-negative bacterium) consists of a lipopolysaccharide outer membrane, with a thin peptidoglycan layer that rejects adsorption of carbon dots [56]. The antimicrobial activity observed in this study is primarily attributed to the migration of active ingredients from the film matrix. All the above discussions show that P-CDs have enormous potential in terms of food contamination by bacteria.

### 3.2. Characterization of the Film

#### 3.2.1. Morphological Analysis

**Figure 5:**
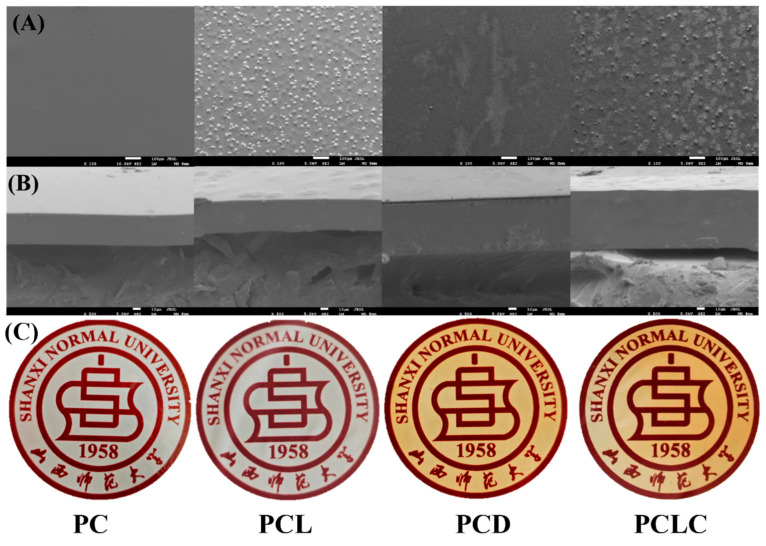
Surface (**A**) and cross-section (**B**) SEM images of PC, PCL, PCD, and PCLC films, along with an image of the surface’s appearance (**C**). Values are expressed as mean ± standard deviation (*n* = 3).

As shown in Figure 5, SEM images and visual images of the films are presented. The PC film surface appears relatively smooth and flat, with no obvious particles or protruding structures, exhibiting good uniformity and a relatively dense surface morphology. This is in agreement with the results of Ma et al. [57]. Numerous uniformly distributed fine granular structures can be observed on the PCL film surface, attributed to the presence of L-Cys [58]. The PCD film surface appears slightly rough with irregular textures, likely resulting from P-CD aggregation during hot-air drying. However, the PCLC film exhibits increased surface roughness and protrusions, indicating that the incorporation of L-Cys and P-CDs successfully alters the film’s microstructure. Cross-sections of the films are depicted in Figure 5B. The PC film cross-section still has a comparatively smooth structure, whereas the granular and rough texture of the cross-sections of the other three films is reflected in the surface appearance in Figure 5A. The film thickness increases with the addition of P-CDs and L-Cys. Visually (Figure 5C), both PC and PCL films exhibited transparency. The PCD and PCLC films displayed a pale-yellow hue due to the incorporation of P-CDs. However, the addition of L-Cys imparted a frosted appearance to both PCL and PCLC films, increasing their opacity.

#### 3.2.2. Chemical Structure Analysis of Composite Films

**Figure 6:**
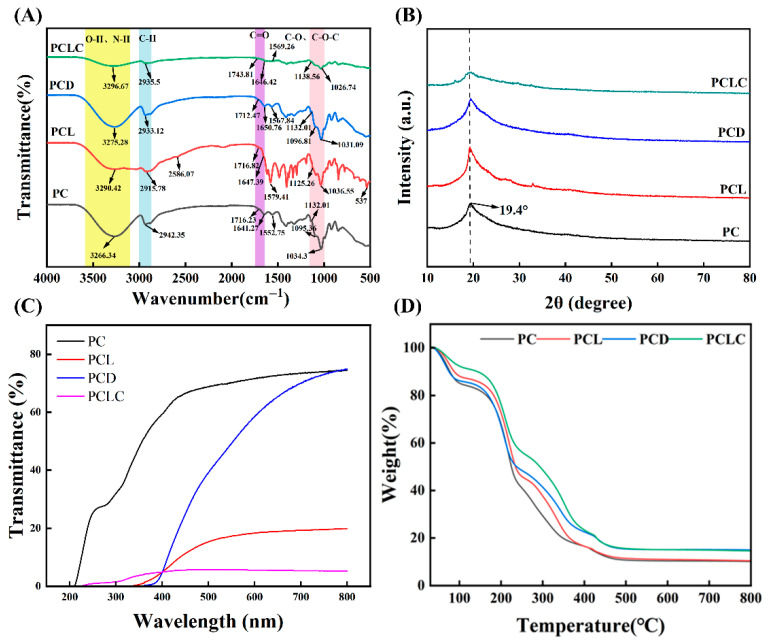
FTIR spectrum (**A**) and XRD pattern (**B**) of the film, along with its light transmittance (**C**) and thermal stability (**D**). Values are expressed as mean ± standard deviation (*n* = 3).

FTIR was used to test the interactions of the chemical functional groups in PVA/CS and L-Cys and P-CDs. Figure 6A represents the FTIR spectra of the various films. A high peak between 3200–3600 cm^−1^ is ascribed to the stretching vibrations of -OH and -NH groups. The maximum at 2900 cm^−1^ is a result of the stretching vibration of -CH groups. Amide I at 1640–1660 cm^−1^ and C=O at 1650–1660 cm^−1^ are identified as the greatest absorbances of the stretching vibration and C=O group, respectively, and amide II and N-H bending vibrations are attributed to the peak at 1550–1660 cm^−1^ [59], which are characteristic of PVA and CS. The absorption peak at around 1030 cm^−1^ (-CO) has been reported to be the result of an interaction between the hybrid film matrix, polyvinyl alcohol (PVA), and chitosan (CS) [60]. Moreover, 1700 cm^−1^ (C=O), 1100 cm-1 (C-C-C), and 1030 cm^−1^ (C-O) vibrations also indicate the presence of PVA in its composition [45]. The PCL film had a weak peak at 2586.07 cm^−1^ and a strong characteristic peak at 537 cm^−1^, which is related to the stretching vibration of the L-cysteine (-SH) sulfhydryl [17], ensuring the incorporation of L-cysteine (L-Cys) into the PCL film. In addition, the lack of typical peaks in the PCLC film of 2550–2600 cm^−1^ can be linked to the reaction of the L-cysteine (-SH) and hydroxyl (-OH) or amino (-NH_2_) groups of polyvinyl alcohol (PVA) and chitosan (CS) [61]. The FTIR spectrum indicates that the doped carbon dot films did not alter the spectral frequency range, affecting only the positions and intensities of characteristic peaks. Since the –OH and –NH_2_ groups on the surface of P-CDs act as hydrogen bond donors, they form intermolecular hydrogen bonds with the –C=O group of the –COOH and the nitrogen atom of the –NH_2_ group in L-Cys. At the same time, the –SH and –NH_2_ groups of L-Cys also act as donors, forming a hydrogen bond network with the –C=O and –C-O-C groups of P-CDs, thereby significantly enhancing compatibility between the two. In an aqueous solution, the –NH_2_ groups of L-Cys readily undergo protonation to form positively charged –NH_3_^+^ ions, while the –COOH, –OH, and other oxygen-containing functional groups on the surface of P-CDs weakly dissociate to produce negatively charged groups (–COO^−^, –O^−^). The attraction between positive and negative charges creates electrostatic interactions, further strengthening the interfacial bonding force. These interactions collectively support the synergistic enhancement of the composite film’s mechanical properties, antioxidant properties, and antibacterial properties. This suggests complete incorporation of carbon dots into the L-Cys and PVA/CS film matrices, forming a film with excellent compatibility [62].

All composite films had their crystalline properties and microstructure studied by X-ray diffraction (XRD), and their results are presented in Figure 6B. All films exhibited a distinct diffraction peak at 19.4°, attributed to the (101) crystal plane of PVA [63], indicating a typical semi-crystalline structure [64]. The addition of P-CDs and L-Cys, respectively, increased the concentration of this diffraction peak, which promptly means that the crystallinity of the composites increased. However, the simultaneous addition of L-Cys and P-CDs resulted in a significant weakening and broadening of the 19.4° peak. This may be attributed to the interaction between the two added substances, disrupting the ordered molecular chain arrangement, resulting in a decrease in crystallinity [65].

#### 3.2.3. Transmittance Analysis

Transmittance analysis was performed by determining the ultraviolet barrier properties of the films by measuring the transmittance for the wavelength range 200–800 nm. The PC film was found to be of high transmittance, as demonstrated in Figure 6C. With the addition of P-CDs and L-Cys, the transmittance at 700 nm decreased from 73.4% (PC) to 5.24% (PCLC), indicating the enhanced UV shielding capability of the PCLC film. This is one of the most significant effects, which may be due to the UV-reducing ability of phenolic substances in pomegranate peel precursors [66], reactive groups in L-Cys (e.g., -SH, -NH_2_), and the conjugated structures of biomolecules. Together, these factors constitute the proposed mechanism for the enhanced UV-shielding performance of the PCLC film; however, the specific nature of their synergistic interaction remains to be verified through further experimental studies.

#### 3.2.4. Thermal Stability

**Figure 7:**
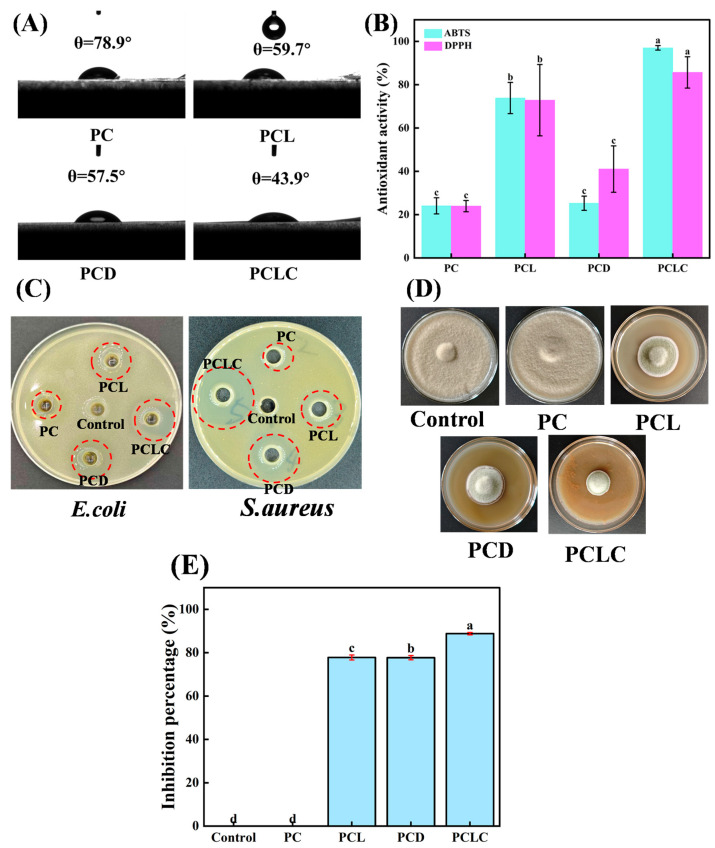
The film’s contact angle (**A**), antioxidant capacity (**B**), antibacterial activity (**C**), and anti-fungal activity (**D**), as well as the inhibition rate of different treatment groups against the *A. alternata* (**E**). Values are expressed as mean ± standard deviation (*n* = 3). Different lowercase letters indicate significant differences at *p* < 0.05 according to one-way ANOVA followed by Duncan’s multiple range test.

Figure 7D shows the thermogravimetric analysis (TGA) curves of PC, PCL, PCD, and PCLC films. All samples exhibit three distinct thermal decomposition weight loss stages. The first stage occurs within the 25–150 °C temperature range, with this initial mass loss attributed to the evaporation of surface moisture and glacial acetic acid [67]. A second decomposition stage was observed between 150 and 250 °C, with PC films undergoing abrupt thermal decomposition at around 280 °C. This is attributed to chitin degradation due to glycosidic bond cleavage and polyvinyl alcohol (PVA) chain dehydroxylation via thermal oxidative decomposition [45]. The third stage occurred between 250 and 480 °C, with the final degradation phase ceasing on average at 480 °C. Overall, the PCLC group exhibited the highest thermal stability among the films, showing a 5% increase in residual mass. This phenomenon likely arises from the formation of hydrogen bonds and thermally stable carbon structures after the incorporation of P-CDs and L-Cys into the PVA/CS film matrix [68]. These structures further hindered water evaporation, thereby delaying the thermal decomposition process.

#### 3.2.5. Analysis of the Mechanical Properties of the Film

In the case of food packaging, the mechanical properties of films are very crucial. Tensile strength testing evaluates their maximum tolerance to external forces, while elongation at break assesses their ductility [69]. As can be observed in Table 1, the mechanical properties of the film, TS (10.53 MPa) and EAB (43.37%) of PCLC were much higher than those of the previous three groups. This improvement in mechanical properties stems from changes in the film composition. Although there was no significant difference in thickness between the PCL and PCLC films, the elongation at break of the PCL film was 31.4% lower than that of the PCLC film. This enhancement may be attributed to the chemical structures formed between L-Cys and P-CDs with polysaccharides, where hydrogen bonding and crystallinity strengthen molecular packing forces [70].

#### 3.2.6. WVP and WCA

Table 1 indicates that the WVP of the PC film is 1.78 × 10^−10^ g·m^−1^·s^−1^·Pa^−1^. The addition of L-Cys, as well as P-CDs, enhanced the WVP up to 2.18 × 10^−10^ g·m^−1^·s^−1^·Pa^−1^, likely due to the hydrophilic groups (–OH, –NH_2_) in L-Cys and P-CDs enhancing the film’s moisture absorption capacity [71]. Water contact angle measurements reflect surface hydrophilicity/hydrophobicity: a larger θ indicates stronger hydrophobicity, while a smaller θ indicates stronger hydrophilicity. As shown in Figure 7A, the PC film exhibits a WCA of 78.9°, indicating relatively strong hydrophobicity. The PCLC film demonstrates the highest hydrophilicity with a WCA of 43.9°, which is attributed to the hydrophilicity of L-Cys and P-CDs reducing the film’s WCA. These results align with those reported by Sul et al. [72].

#### 3.2.7. Antioxidant Activities and Antimicrobial Properties of Composite Films

Figure 7B shows the antioxidant activity of the composite membranes. As evident from Figure 7B, all membranes exhibit antioxidant activity, with the PC membrane displaying the lowest antioxidant activity. This antioxidant phenomenon is attributed to the hydroxyl and amino groups present in chitosan [73]. The incorporation of L-Cys and P-CDs showed the highest antioxidant activity of the PCLC membrane (97.21% with ABTS and 85.76% with DPPH), which is a 305.37% and 260.48% increment, respectively, over the control (PC). This may be attributed to the reductive sulfhydryl groups of L-Cys [74] and functional groups, i.e., carboxyl and hydroxyl groups in P-CDs [75]. Notably, membranes supplemented with L-Cys exhibited slightly higher ABTS radical scavenging rates than DPPH. This discrepancy likely arises because the thiol group in L-Cys efficiently scavenges water-soluble radicals through pathways such as hydrogen atom transfer and electron transfer, whereas DPPH is lipophilic, causing L-Cys to disperse unevenly within it.

The antimicrobial efficacy of packaging materials is crucial for maintaining food freshness and inhibiting microbial growth. Figure 7C,D illustrate the effects of disparate composite films on bacteria *(E. coli* and *S. aureus*) and fungi (*A. alternata*) that possess antibacterial activity. The PCLC group has been proven to have a significant amount of antibacterial activity towards *E. coli* and *S. aureus,* as demonstrated in Figure 7B. All treatments had a high level of antibacterial activity against Gram-positive bacteria when compared to Gram-negative bacteria. The reason for this is that *E. coli*, which is Gram-negative, has a multifaceted outer membrane layer that hinders the process of antimicrobial agents entering the cell [76]. *Alternaria alternata* is one of the primary pathogens causing postharvest rot in cherry tomatoes [77]. Figure 7D illustrates the antimicrobial effects of different composite films against *A. alternata*. No significant inhibition zones were observed in the PC and control groups, while the PCLC group exhibited the smallest colony diameter and strongest anti-fungal capability. Figure 7E shows that the inhibition rate in the PCLC group was significantly higher than in the other groups, exceeding that of the control group by 88.8%.

### 3.3. Application of Composite Films in Cherry Tomato Preservation

#### 3.3.1. Appearance and Rot Rate of Cherry Tomatoes

**Figure 8:**
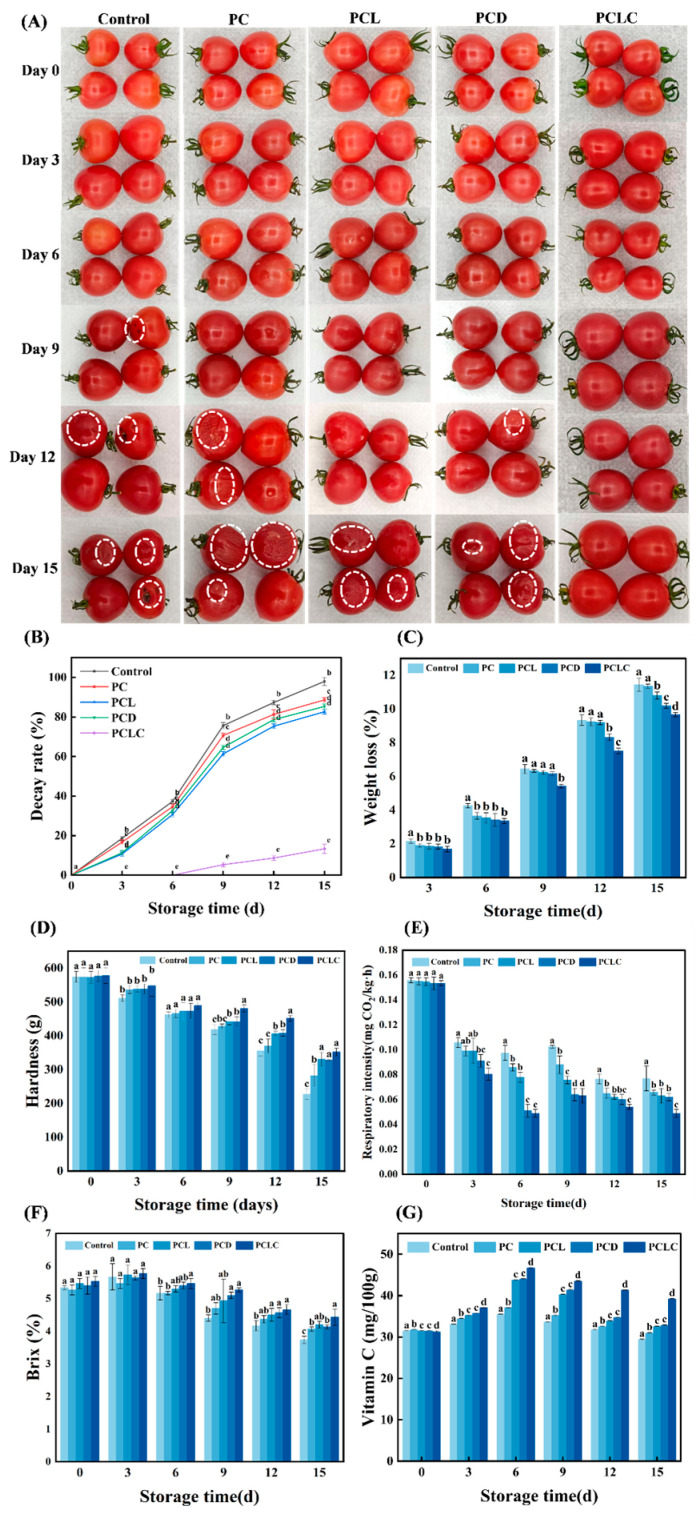
Appearance (**A**); decay rate (**B**); rate of weight loss (**C**); hardness (**D**); respiratory intensity (**E**); solubilized solids contents (**F**); and vitamin C contents (**G**) of cherry tomatoes for the various treatment groups during storage. Values are expressed as mean ± SD (*n* = 3). Different letters indicate significant differences at *p* < 0.05.

Given cherry tomatoes’ high water content, susceptibility to wilting, and vulnerability to microbial infection leading to spoilage, their surface condition serves as a critical indicator for quality assessment [78]. The appearance of cherry tomatoes from each treatment group during storage is shown in Figure 8A. In the early storage phase, the tomato skins of all groups appeared smooth, plump, and without wrinkles. However, as storage time extended, black spots appeared on the surface of control tomatoes by day 9. On the 12th day, the count of black spots had grown in the control group, whereas in the PC and the PCD groups, shriveling and slight softening were identified, respectively. By day 15, the PCL group displayed significant shriveling and minor pathogen infection. The PCLC group had the best appearance throughout the entire storage period, and the shelf life of PCLC was 6 days longer than that of the control group. Figure 8B presents the rate at which cherry tomatoes were decaying over time. All groups showed a higher rate of decay with time. The rate at which the PCLC group (13.59%) decayed on day 15 was only 13.85% of the control group (98.14%). This shows that the addition of L-Cys and P-CDs was useful in increasing the storage time of cherry tomatoes.

#### 3.3.2. Weight Loss Rate and Hardness

Figure 8C,D indicate that the rate of weight loss in cherry tomatoes was usually accelerated, and the hardness was reduced. The rate of weight loss in the coated group during storage was consistently low compared to the non-coated group; this was explained by the natural barrier characteristics of the coating [79]. The rate of weight loss continued to be significantly lower in the PCL and PCD groups after 15 days of storage compared to the control group. The PC group was shown to be able to inhibit fruit weight loss well within the initial 6 days of storage, but there was no significant difference between the PC and the control group after the 6 days. This can be explained by the fact that PC films have very weak moisture barrier properties, which are in line with the previously analyzed WVP results. At day 15, the PCLC group exhibited the lowest weight loss rate, 15.63% lower than the control. This may result from the crystal structures of L-Cys and P-CDs creating more complex water pathways within the composite film, thereby reducing weight loss. As evident in Figure 8D, the firmness of tomatoes steadily declined throughout the storage period among all the treatment groups, which is in line with the natural postharvest process of tomatoes, which dry up and soften. The control group exhibited a significantly faster rate of decline in firmness compared to other treatments, while the PCLC group maintained consistently higher firmness levels, indicating that this treatment effectively delayed tomato softening.

#### 3.3.3. Respiratory Intensity

After harvest, cherry tomatoes consume their own nutrients through respiration to provide energy for the fruit. Higher respiration intensity accelerates nutrient depletion and tissue aging, resulting in poorer storage quality [80]. According to Figure 8E, all cherry tomato groups had reduced respiratory intensity during storage, with the control group having the highest rate of respiratory reduction. The PCLC group demonstrated significantly lower respiratory intensity than the other four treatment groups. After 15 days, the respiratory intensity of the PCLC-treated group was reduced by 63.64% of the control group. This likely resulted from the creation of a controlled internal environment and selectively permeating carbon monoxide and O_2_ between the fruit interior and exterior, thereby reducing respiration and transpiration rates and delaying fruit ripening [81].

#### 3.3.4. Soluble Solids and Vitamin C Content

Figure 8F illustrates changes in the soluble solids content during cherry tomato storage. Between days 9 and 15, the content of the soluble solids in the control group declined significantly, with a significant difference between the control, PCD, and PCLC groups. Among these groups, the PCLC group exhibited the highest soluble solids content, effectively preserving tomato sweetness and quality. Figure 8G illustrates the changes in vitamin C (VC) levels in cherry tomatoes during storage. Generally, there was an increase in VC levels followed by a decline, with treatments helping to preserve the VC content. As of day 15, the VC concentration of cherry tomatoes in the PCLC group was increased, respectively, by 33.06 and 26.8% compared to the control group and PC coating group. This means that the PCLC composite coating was successful in curbing the depletion of nutrients in stored cherry tomatoes.

In summary, the PCLC composite coating treatment preserves the soluble solids and vitamin C content of cherry tomatoes, inhibits fruit respiration, reduces weight loss, and lowers the rate of firmness loss and spoilage. This extends the shelf life of cherry tomatoes and holds significant commercial importance for reducing postharvest losses, extending distribution cycles, lowering storage and transportation costs, enhancing market competitiveness, and meeting the demand for green fresh food supplies.

## 4. Conclusions

This study successfully synthesized carbon dots (P-CDs) from waste pomegranate peels. P-CDs were uniformly incorporated with L-cysteine (L-Cys) into the polyvinyl alcohol–chitosan (PVA/CS) composite matrix, thereby forming a bioactive coating. The physical and chemical characteristics of this film were examined, and its usage in cherry tomato preservation was analyzed in order to identify the impact of preservation. The findings obtained show that synthesized P-CDs are spherical in shape and well dispersed. It is worth noting that the addition of P-CDs and L-Cys also contributes greatly to the antioxidant activity of the composite film (97% using the ABTS method; 85.69% using the DPPH method), the mechanical property of the PC composite film (tensile strength, increasing from 3.98 MPa to 10.53 MPa, and elongation at break, increasing from 21.77% to 43.3%), and ultraviolet shielding (PCLC ultraviolet shielding). In addition, the diameter of the inhibition zone was assessed against E. coli (7 mm), S. aureus (12 mm), and A. alternata (28 mm). Freshness preservation tests on cherry tomatoes demonstrated that the PCLC composite coating effectively extended shelf life from 9 to 15 days while slowing nutrient depletion in the fruit. However, the film’s hydrophobicity requires further optimization.

On the whole, the newly developed coating material exhibits preliminary biocompatibility and shows potential application prospects in the preservation of fruits and vegetables with a high-value utilization of agricultural waste, in line with the concept of green chemistry and sustainable development. Notably, this study only verifies the preliminary biocompatibility of the coating, and its actual food safety applicability for commercial food preservation still requires in-depth and comprehensive verification through subsequent studies (including food contact migration tests, long-term biocompatibility assessments, and toxicological evaluations).

## Figures and Tables

**Table 1:** The thickness, water vapor permeability, tensile strength, and elongation at break of films.

Film Type	Thickness (μm)	WVP (×10^−10^ gm^−1^s^−1^Pa^−1^)	Ts (MPa)	EAB (%)
PC	50.3 ± 0.57 ^b^	1.78 ± 1.28 ^bc^	3.98 ± 0.009 ^c^	21.77 ± 0.054 ^c^
PCL	95.67 ± 1.52 ^a^	2.78 ± 2.12 ^ab^	2.92 ± 0.089 ^d^	11.97 ± 0.36 ^d^
PCD	42.16 ± 1.46 ^c^	7.21 ± 5.86 ^bc^	9.95 ± 0.042 ^b^	31.79 ± 0.13 ^b^
PCLC	97.6 ± 1.25 ^a^	3.96 ± 1.83 ^a^	10.53 ± 0.013 ^a^	43.37 ± 0.74 ^a^

Values are presented as the mean ± standard deviation (*n* = 3). Different lowercase letters in the same column indicate significant differences at *p* < 0.05.

## Data Availability

The original contributions presented in the study are included in the article; further inquiries can be directed to the corresponding authors.
